# Assessment of Quality Indices and Their Influence on the Texture Profile in the Dry-Aging Process of Beef

**DOI:** 10.3390/foods11101526

**Published:** 2022-05-23

**Authors:** Viorica Bulgaru, Liliana Popescu, Natalia Netreba, Aliona Ghendov-Mosanu, Rodica Sturza

**Affiliations:** Faculty of Food Technology, Technical University of Moldova, 9/9 Studentilor St., MD-2045 Chisinau, Moldova; liliana.popescu@tpa.utm.md (L.P.); natalia.netreba@tpa.utm.md (N.N.); aliona.mosanu@tpa.utm.md (A.G.-M.); rodica.sturza@chim.utm.md (R.S.)

**Keywords:** beef, aged meat, sensory and physicochemical indicators, texture parameters, information analysis

## Abstract

The aim of this study was to investigate the effects of the dry-aging method on the sensory properties, chemical composition, and profile parameters of the texture of beef obtained from local farms. The qualitative characteristics of the beef were investigated for five samples, respectively, fresh meat, and dry-aged beef for 14, 21, 28, and 35 days, in aging rooms with controlled parameters: temperature (1 ± 1 °C), relative humidity (80 ± 5%), and air circulation speed (0.5–2 m/s). During the dry-aging period, there was a decrease in humidity by about 6.5% in the first 21 days, which allowed the concentration of fat, protein, and total collagen content. The dry-aging process considerably influenced the pH value of the meat, which, in the second part of the dry-aging process (14–35 days), increased from 5.49 to 5.66. These values favored the increase by 37.33% of the water retention capacity and the activation of the meat’s own enzymes (calpain, cathepsin, collagenase). This influenced the solubilization process of proteins and collagen, thus contributing to the improvement of the texture profile. Because variations in organoleptic and physicochemical parameters occurred simultaneously during dry-aging and storage, the method of analyzing the information was applied. Mutual information on the influence of physicochemical indicators on the texture profile parameters was followed, a factor of major importance in the consumer’s perception. The degree of influence of soluble proteins, sarcoplasmic and myofibrillar proteins, fats, and soluble collagen content on the texture profile parameters (hardness, cohesiveness, springiness, gumminess, and chewiness) of the dry-aged beef for 35 days was established. These investigations allowed the optimization of the beef dry-aging technological process in order to obtain a product with a sensory profile preferred by the consumer.

## 1. Introduction

Meat has been and is an indispensable product of the human diet, both as a food in itself, and as an essential ingredient in many other foods, due to its chemical composition and valuable biological value [1,2,3]. According to the Organization for Economic Cooperation and Development (OECD), the world production of beef in 2021 increased by 4.8 million tons compared to the level of 2012, which amounted to 6.8%. The main counterparties in the beef market are the USA, Brazil, and China, which provide more than 40% of the world production [4]. The production of beef in the European Union countries over the past decade has slightly decreased by an average of 1.5%, and holds a more stable position, providing about 10% of the world beef production [5].

Meat is mostly the muscle tissue of an animal, with a complex chemical composition [6,7,8].

Beef has a protein content of between 26 and 31% [9,10], which is the main constituent of the structure of the meat product [9]. Actin and myosin (myofibrillar proteins) represent about 2%, soluble sarcoplasmic proteins constitute about 6, and 2% are the connective tissues—collagen and elastin, which cover the structural protein [11]. Collagen differs from most other proteins in that it contains the amino acids, hydroxylysine and hydroxyproline. Elastin, also present in connective tissue, has less hydroxylysine and hydroxyproline. Thus, the protein content of meat rich in connective tissue is lower than that of meat without connective tissue. The presence of connective tissue makes the product harder and with low nutritional and economic value [6].

Beef is appreciated for its important content of macronutrients in ensuring a healthy and balanced diet. The content of proteins, proteolytic enzymes together with the speed of muscle contraction (beef—slow contraction), and the type of metabolism (oxidative for beef) determine the variation of the muscle-type speed maturation. During the aging process, the meat’s own enzymes contribute to the improvement of the meat’s quality, achieving the tenderization of the meat by their action on the myofibrillar and sarcoplasmic system. Calpain and cathepsin mainly degrade myofibrillar proteins, resulting in increased soluble protein content. Some cathepsins (B, L, H) together with the multifunctional system enzymes (proteasome, prosome) weaken the connective tissue [12].

The beef industry is constantly looking for new ways to meet consumer demand for high-quality products. The taste of beef is described as a combination of three factors: tenderness, aroma, and juiciness, and the meat humidity has an important role in determining these factors. The combination of these factors allows the consumer to perceive the taste of the product [6,13,14,15].

In this context, in order to obtain high characteristics of tenderness, juiciness, and consistency, beef can be subjected to the ageing process. The aging of meat is defined as a process which naturally enhances the taste and tenderness of the whole carcass or its parts at refrigerated temperatures [16].

In general, there are two forms of beef-aging techniques: wet and dry, which result in flavor development and more tender meat. Dry- and wet-aging methods are widely applied for the aging of meat [17,18]. When beef is wet-aged, it is put in a vacuum-sealed package and stored in a controlled environment for a specific period of time, in vacuum packages at 1–3 °C for a couple of weeks. The length of treatment varies between 3 to 90 days [19].

Dry-aging is the process of placing the unpackaged beef carcasses in an aging room and leaving them to age for several weeks at controlled parameters [20,21]. The key effect of dry-aging is to concentrate the flavor that can only be described as “dry-aged beef” [17,22,23]. During the dry-aging process, the juices are absorbed into the meat, and the chemical breakdown of protein and fat constituents occurs, which results in a more intense nutty and beefy flavor. During aging, the beef’s natural enzymes break down the proteins and connective tissue in the muscle, which leads to more tender beef [22,24,25]. The dry-aging process takes place in aging rooms with predetermined parameters: temperature (1–3 °C), and 70–85% humidity, for a minimum of 21–28 days on average, without the application of any protective packaging process. In this process, the unique aroma and tenderness occur due to enzymatic and biochemical changes in the meat [16].

There are different opinions in the industry about desirable flavor. Most people agree that dry-aging results in a unique flavor. However, people not familiar with dry-aged beef often describe it as slightly “musty” in flavor when eaten for the first time. The study of [26] showed that dry-aging resulted in a more intense beef flavor, more intense color, and less moisture content compared with aging “in the bag”.

The aim of this research is to study the influence of the dry-aging process stages on the sensory, physicochemical, and texture indices of dry-aged beef.

## 2. Materials and Methods

### 2.1. Materials

For analysis, Simmental beef was cut and sliced from the carcass: T-bones and Ribeye, freshly received from the local farm, Bio Energy Farm, Firladeni, Hincesti, Republic of Moldova, which meet the quality requirements set out in [27].

Beef was dried during 35 days in the aging room with controlled parameters: temperature (1 ± 1 °C), relative humidity (80 ± 5%), and air circulation speed (0.5–2 m/s).

### 2.2. Chemical Materials

The hexane reagent (>99%), 2-propanol, (≥99%), buffer solution, pH 6.00, chloramine T-reagent, hydrogen peroxide, (35%), hydrochloric acid (37%), phosphate buffer pH 7.2, 4-(Dimethylamino) benzaldehyde (98%), perchloric acid puriss. (70.0–72.0%), and trans-4-hydroxy-L-proline (≥99%) were provided by Sigma-Aldrich (Schnelldorf, Germania); the Kjeldahl mineralization Catalyst CX 5.5 GR was purchased from VWR Chemicals (Lutterworth, UK); and **v**receiver TKN (Bromocresol green and Methyl red) was obtained from Velp Scientifica (Usmate, Italia).

### 2.3. Methods

The determination of meat quality indices was performed on the 14th, 21st, 28th, and 35th day of dry-aging.

#### 2.3.1. Sensory Analysis of Fresh and Aged Meat by Comparison with Unit Scoring Scales

The sensory properties of the meat samples (Table 1) were analyzed by standard ISO 6658:2017 [28]. Appearance, color, consistency, and flavor were assessed using the 5-point system by an expert panel of nine trained specialists in the food industry field. The 5-point assessment system includes the following scores: 5—very good; 4—good; 3—satisfactory; 2—poor; 1—bad; and 0—very bad.

#### 2.3.2. Sensory Analysis of Fresh and Aged Meat by Constructing the Sensory Profile

The method is based on identifying the descriptors, measuring the perceived sensory intensity for each descriptor chosen, and, with the help of a quantified descriptors set, constructing a flavor sensory profile that characterizes the meat sample. Panelists (*n* = 9) were food industry specialists from the Department of Food Technology, Technical University of Moldova, with experience in performing sensory analysis of food products. The beef for sensory analysis was cut into pieces sized 1.27 × 1.27 × 2.54 cm and randomly identified with two-digit codes. The panelists evaluated the sensory attributes for flavor on a five-point scale, with ”0” being equivalent to the absence of sensation for the analyzed descriptor, and “5” being a very strong sensation for the analyzed descriptor. The flavor sensory profiles of the aged meat samples are presented in the form of a diagram [28,29]. 

#### 2.3.3. Moisture Content

This parameter was determined according to gravimetric method, based on the weight loss of the analyzed sample to constant mass, due to water evaporation by heating in an oven at a temperature of up to 130 °C [30].

#### 2.3.4. Fat Content

The Soxhlet method was used to determine fat content, based on the fat extraction from the dehydrated sample using organic solvents in the Soxhlet apparatus [31]. 

#### 2.3.5. Total Protein Content

This parameter was measured by the Kjeldahl method—digestion of a sample with concentrated sulfuric acid, which converts organic nitrogen into ammonium ions, under the catalytic action of copper (II) sulphate; the process of alkalization, distillation of excess released ammonia solution of boric acid, titration with hydrochloric acid of ammonia combined with boric acid, and calculation of nitrogen content takes place [32].

#### 2.3.6. Protein Solubility

To determine the solubility of the protein, the method described by [33], slightly modified by Ismail, et al., was used [9]. Sarcoplasmic proteins were extracted from 1 g of muscle (minced) using 20 mL of ice-cold 0.025 M potassium phosphate buffer (pH 7.2) by homogenization at 11,000 rpm. The homogenized samples were kept under refrigerated conditions for 20 h at 4 °C, and then centrifuged at 3000 rpm for 15 min (4 °C). The supernatant was filtered through a paper filter into a 250 mL graduated beaker. The extraction process was repeated four times, and then the contents of the graduated beaker were leveled with phosphate buffer solution (pH 7.2). In addition, the protein concentration was measured using the Kjeldahl method.

Total protein solubility was similarly determined in a 1.1 M KI, 0.1 M potassium phosphate buffer (pH 7.2).

Myofibrillar protein solubility was calculated by the difference in the solubility of total and sarcoplasmic proteins.

#### 2.3.7. Total Collagen and Collagen Solubility

Total collagen was determined according to ISO-3496 [34], with modification after Ismail, et al. [9]. Four grams of meat was hydrolyzed with 30 mL of 3.5 M H_2_SO_4_ for 16 h at 105 °C. The hydrolysate was filtered, and the solution was diluted to a volume of 500 mL with distilled water. The diluted solution (1 mL) was pipetted into a 100 mL graduated cylinder and filled to the mark with distilled water. The final dilution (2 mL) was pipetted into a test tube, and 1 mL oxidation solution (1.41 g chloramines-T reagent and 100 mL pH 6.0 buffer solutions) was added and left at room temperature for 20 min. Color reagent (1 mL) (dissolving 10 g 4-dimethylamino benzaldehyde in 65 mL 2-propanol and 35 mL perchloric acid) was added and mixed. The tube was capped and then placed in a water bath at 60 °C for 15 min. After cooling, the absorbance of solutions was measured at 560 nm with UV-Vis spectroscopy. A standard calibration curve was carried out from hydroxyproline at concentrations of 0, 1.2, 2.4, 3.6, and 4.8 μg hydroxyproline/mL. The collagen content was calculated from hydroxyproline content using the coefficient 8. 

The soluble collagen in the cooking loss was centrifuged at 3000 rpm, at 4 °C for 30 min. Supernatant (5 mL) was hydrolyzed, diluted with distilled water (first to 100 mL, then to 50 mL), and measured for hydroxypropylation concentration as the described total collagen. Collagen solubility (%, soluble collagen in the cooking loss) is determined by the formula:

(1) $$\%\;\mathrm{Collagen}\;\mathrm{solubility}=\frac{C_{s}}{C_{t}}\cdot 100$$

where: $C_{s}$—soluble collagen in cook loss, g; $C_{t}$—total collagen of raw meat, g.

#### 2.3.8. Determination of the Meat pH

Active acidity of the meat was performed with the pH meter Titroline 5000 at 20 °C.

#### 2.3.9. Determination of Water Holding Capacity (*WHC*)

The minced meat samples (20 g) were mixed with 30 mL of 0.6 M sodium chloride in a centrifuge tube. The tubes were kept at a refrigeration temperature of 4 ± 1 °C for 15 min, and then homogenized for one minute and centrifuged at 3000 rpm for 15 min. The supernatant was measured and the *WHC* was expressed as a percentage, according to [35], using the following formula (2):

(2) $$WHC=\frac{V_{2}-V_{1}}{V_{1}\;\cdot \;100}$$

where: *WHC*—water holding capacity, %; $V_{1}$—NaCl volume to centrifugation, mL; $V_{2}$—NaCl volume after centrifugation, mL.

#### 2.3.10. Texture Profile Analysis

Texture profile analysis was performed with a texture analyzer (Stable Micro Systems TA.HD plus C, UK). To determine the textural properties of the meat samples (40 mm × 40 mm × 20 mm) (hardness, cohesiveness, springiness, gumminess, resilience, chewiness), a double compression test was performed using spherical stainless 5 mm (P/5s), and respecting the following steps and parameters: pre-test speed—100 m/s; test speed—5 m/s; post-test speed—5 m/s, cell load—5 kg [36].

### 2.4. Mathematical Modeling

Information analysis of experimental data was performed in the MATLAB program (MathWorks, Inc., Natick, MA, USA), which allows us to establish the influences between the various quantities measured during the tests. Names of the physicochemical indicators are given in the rectangles of the graph, and the values of mutual information, measured in bits, are mentioned on the arrows of the graph. The more pronounced the influence between the various measured quantities, the higher the value of the bit [37].

### 2.5. Statistical Analysis

All calculations were performed using Microsoft Office Excel 2007 (Microsoft, Redmond, WA, USA). Data obtained in this study are presented as mean values ± the standard error of the mean calculated from three parallel experiments. The comparison of average values was based on the one-way analysis of variance (ANOVA) according to Tukey’s test at a significance level of *p* ≤ 0.05, using the Staturphics program, Centurion XVI 16.1.17 (Statgraphics Technologies, Inc., The Plains, VA, USA).

## 3. Results and Discussions

Aging is one of the oldest methods used to enhance the tenderness and taste of meat. Meat tenderness is caused by the enzymes activity that cause proteolysis of myofibrils and connective tissue proteins. The proteolytic effect of the calpain system and cathepsin is a determining factor for the tenderization of meat [38,39]. In the maturation process, it is known that the mechanism that increases taste and aroma is caused by increasing the density of flavor components (stearic acid, linoleic, palmitic, oleic acid, glutamate, carnosine, inosine monophosphate) of tissues by water loss [40,41], and the mechanism that causes meat to be tender is caused by the distortion of connective tissue by natural enzymes in muscle tissue [16,42].

For the analyzed aged meat samples, the physicochemical and sensory indices were determined (Table 2).

Beef quality indicators (Table 2) are affected by pre- and post-slaughter factors, and genetic factors [43]. The palatability of the meat is influenced by the flavor, juiciness, and tenderness of parameters that directly affect visual consumer perceptions, as well sensory parameters [43,44,45].

**Figure 1:**
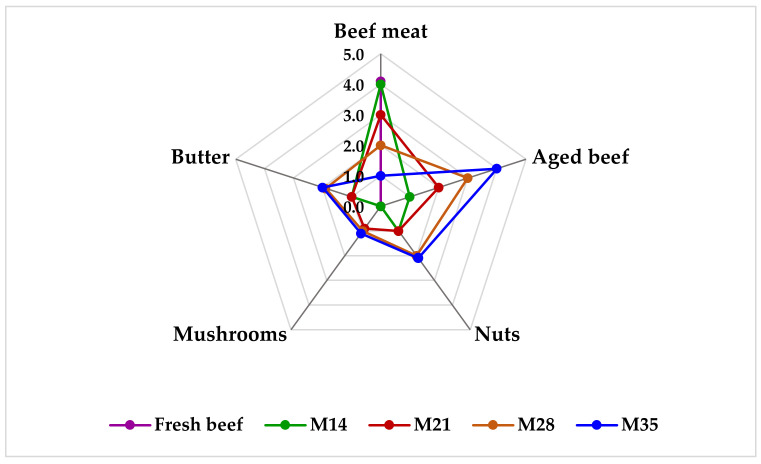
Evolution of the flavor profile of dry-aged meat for 35 days.

Aged beef obtained by the natural dry-aging process, in compliance with the proposed parameters of temperature, humidity, and speed of air circulation, is characterized by a moist surface, elastic muscle tissue, and regular edges, without deep cuts. An important quality parameter is beef color, because of its influence on the consumer’s purchase and consumption decision, as well as an important feature in assessing the degree of meat-aging [46]. The color of the meat varied according to the aging period, from light red in fresh meat (4.30 points) to dark red in the case of the M35 sample (5.00 points). The intensity of the specific odor of beef decreased with the increase of the aging period, and after 35 days, it was no longer perceived; at the same time, the pleasant flavor of the aged meat intensified, and the appreciation score increased for dry-aged beef compared to the value given to the fresh beef, from 4.00 to 5.00 points. The most appreciated by the panelists were the dry-aged beef samples for 28 and 35 days, characterized by a darker red color and balanced flavor, with an elastic, firm consistency. The process of dry-aging entails the change of aroma precursors, and contributes to the concentration of the aged meat flavor due to the accumulation of aliphatic amino acids, peptides, inosines, and sugars obtained from carbohydrates that give a sweet taste. All these newly formed substances, especially under the action of calcium-dependent enzymes, give the aged meat an aroma of nuts, butter, and a slight taste of mushrooms. Studies showed that the flavor of the meat changes from the 14th day of aging and intensified as the aging period increased (Figure 1). Similar results were described by the authors in their works [22,47,48]. Cho, et al. [49] showed that the sensory quality of fresh and dry-aged beef for 20, 40, and 60 days increased with increasing aging time. Aging for 60 days showed different values for tenderness with aspects of improvement (essential changes are observed even after the first 20 days of aging), and the intensification of flavor, juiciness, and overall acceptability, especially at 40 and 60 days of aging. Increased values for texture, taste, and aroma were also obtained by Lee, et al. [50] for beef aged for 21 days at 2 °C. 

The authors argue that the improvement in sensory quality is due to the formation of metabolites associated with taste, aroma, and consistency (listed above), which are formed in the process of dry-aging, and compounds that are practically not formed, or are formed in small quantities when other meat aging methods are used. Laster, et al. [51] categorized meat obtained by dry-aging as a premium product. Kim, et al. [52] claimed that most metabolites characteristic for improving the meat sensory quality are formed in the process of dry-aging.

During the period of beef dry-aging, the moisture content tended to decrease, by 6.30% after 21 days of aging, and 10.5% after 35 days. The decrease in moisture during the meat dry-aging is a normal process, which can reach up to 10% in the first 21 days of aging, which allows a concentration of the basic meat components, which greatly influences the formation of the specific beef aroma. The results showed that there was an obvious decrease in beef moisture in the first 21 days of aging, followed by a lower decrease in humidity. Similar results have been obtained by Kahraman, et al. and Cho, et al. [16,49]. Cho, et al. showed that this could be related to the capacity of muscle contraction, which is minimal at low temperatures, and the intensity of the dehydration of the surface layers at the beginning of the aging period. So, the decrease in moisture was up to 10% for 60 days of aging, which are close results to those obtained in the present work, possibly due to the fat coating that protected against excessive water evaporation, as well as the crust that formed on the surface of the meat that was subjected to the aging process [49]. In the case of the formation of a thicker crust on the meat surface, the water losses were also lower, as Smith, et al. showed in their paper [53].

The acidity value is an important indicator in directing the evolution of the physicochemical transformations in the meat during the aging period. However, the acidity of the meat is most often expressed by the pH value [54].

The meat pH value slightly increased, by about 3% from 5.50 to 5.66 during 35 days of aging, values that favor the activation of calpain by releasing Ca^2+^ ions from the sarcoplasmic reticulum and mitochondria when the level of ATP is practically zero. These values also fall within the pH range of cathepsins, which degrade myosin and actin in muscle tissue [12]. Moreover, other research, such as Lee, et al. [55] showed an increase in pH value (5.51–6.16, 5.72–5.94 for meat obtained from different producers) during dry-aging for 63 days, and Obuz, et al. [56] demonstrated that the dry-aged meat pH value increased compared with the wet-aged meat for a similar period of 23 days (5.47 vs. 5.45), possibly due to the formation of nitrogenous compounds in the proteolysis process during aging.

Water-holding capacity (*WHC*) is the force with which meat proteins retain part of their own water and part of that which is added under the action of an external force (pressing, cutting, etc.) [57]. For meat processing, it is known that the *WHC* of fresh meat influences its technological quality and the processing efficiency [38]. If myofibrillar proteins bind enough water, they become water soluble, and the solubilization of the myofibrillar proteins is essential for the formation of an emulsion or matrix for the fat encapsulation also [58].

In muscle tissue, the *WHC* of meat proteins is generally favored at a higher pH. As such, this statement explains the obtained results, which denoted an increase in *WHC* of 37.3% (14 and 21 days are with higher increase) with the increasing of the aging period and pH value.

The same dependence was obtained by Lee, et al. [55], where the *WHC* changed according to the pH value, having a growing tendency, and where the beef *WHC* value was greatly influenced by the parameters of the dry-aging process (temperature, relative humidity, and air circulation speed).

During the dry-aging process, the chemical composition—fat, protein, and collagen—of beef did not undergo essential changes. Modifications in their quantitative content depended on the decrease in moisture content in the product, a hypothesis supported by other authors [49]. Kim, et al. and Iida, et al. [52,59] claim that the concentration of protein and fat content increases insignificantly due to the decrease in moisture content, and that their degradation during dry-aging contributes to the formation of the dry-aged beef taste and flavor.

The results obtained for the fat meat content showed an increase of 48.4% for the dry-aged meat during 35 days. The aging conditions and the crust formation on the meat surface during aging protect the meat lipids from oxidation processes, and the observance of the sanitary-hygienic conditions in the aging rooms prevent the undesired lipolysis processes. Fat is the most variable component of meat. It is important for the formation of flavors, texture, and juiciness. It also affects the product shelf-life [17,25,52,59]. Lee, et al., for beef samples obtained from different producers, showed increasing values for fat content: for sample A—an increase of 27.5% for 21 days of dry-aging; and for sample C, an increase of 45.2% for 28 days of dry-aging [55]. Cho, et al., for beef samples dry-aged for 60 days at a temperature of 2 °C and a relative humidity of 85%, without monitoring the speed of air circulation, showed an increase in fat content of almost 20%, which was possible due to the insignificant decrease of moisture content (about 4%) [49].

The total collagen content was not significantly affected by the dry-aging process. The total amount of collagen during aging increased by about 26.8%, and the content of soluble collagen also increased (by almost 3 times). This increase was due to the action of lysosomal glycosidases that facilitate the action of cathepsins in the degradation of the fundamental substance of connective tissue and collagenase. Among metal proteinases, interstitial collagenase causes the weakening of intramuscular collagen fibers and fibrils (from the perimysium and endomysium), increasing the amount of soluble collagen. [60]. Cho, et al. [49] showed a small decrease in total collagen content for beef samples that were dry-aged for 60 days at a temperature of 2 °C and a relative humidity of 85%, due to a small decrease in humidity content. Colle, et al. [61] showed that there were no major differences between the amount of total collagen and soluble collagen during beef dry-aging (loin muscle), possibly because the study was performed on dry-aged beef for a shorter time (14 days).

Moreover, the content of soluble proteins and soluble collagen in aged meat could be influenced by the aging process factors of variation: factors related to species and animal (within the same species or the same animal: age, sex, race, fattening status); factors related to the type of muscle considered, which divide muscle fibers into type II (rapid contraction/glycolytic metabolism) and type I (slow contraction/oxidative metabolism) [12,62].

Protein is the basic component of meat and meat products, in terms of nutritional value. The functions of meat proteins are nutrition, texture, color, and *WHC*. During the dry-aging period, the total amount of protein changed non-essentially, decreasing by about 7.50%, due to the increase of the total dry matter content in the meat, which is similar to results obtained by other authors [49,52,62]. Kim, et al. [62] obtained a slight increase in the protein content of dry-aged beef (1 °C, 0.5 m/s, 85% relative humidity, 30 days), by about 1%. Cho, et al. [49] showed an increase in protein content of 5–6% during 60 days of dry-aging, and Lee, et al. [55] demonstrated an increase in protein content of 4.0–7.5% when the beef was dry-aged for 42 days.

The total soluble protein content increases during dry-aging due to the activity of endogenous proteases present in meat [63]. The conditions of the aging process (pH value, the release of Ca^2+^ ions from the sarcoplasmic reticulum) favor the activity of the meat’s own enzymes, especially calpain and cathepsins, which cause the degradation of proteins to peptides [12]. In this way, the proportion of activated free calpain increases from 15 to 97% of the total calpain. Free calpains also make the meat tender by their action on the myofibrillar and sarcoplasmic system. As the pH decreases, the proteolytic activity of the calpain decreases to a minimum at pH~5.5. [14]. Cathepsins (lysosomal proteases) are located in lysosomes and have maximum activity at pH = 4–6 [12].

Therefore, the pH values of the meat during the aging period (pH 5.5–5.66) favor the activity of calpain and cathepsins contributing to the solubilization of sarcoplasmic and myofibrillar proteins. The soluble protein content varies within the limits of 2.61 (fresh beef), 2.98 (M14), 2.99 (M21), 3.0 (M28) and 3.01% (M35). During the dry-aging process, there was an increase of 15.30% for total soluble proteins, an increase of 63.1% for sarcoplasmic proteins (the highest increase being observed in the aging period of 28 and 35 days), and a constant decrease in myofibrillar proteins of 26.6%. Kim, et al. [62] showed sodium dodecyl sulfate-polyacrylamide gel electrophoresis pattern (SDS-PAGE) results of protein samples obtained after the aging of beef loins that significantly affected the percentage intensity of several protein bands (20 to 48 kDa) compared to the control group. Toldra [64] reported that the myosin light chain band (16 to 27.5 kDa molecular weight) intensity of the dry-aged beef protein samples was higher than that of the control sample and wet-aged beef. Claeys, et al. [65] reported that the SDS-PAGE protein separation patterns displayed a range of 3 to 17 kDa proteins in aged beef.

Denatured (solubilized) sarcoplasmic proteins produce porous structures between denatured myofilaments, which results in a gel-like matrix that contributes to better water retention in the structure of meat. The research conducted by [9] regarding the water-holding capacity in meat, even after heat treatment, showed that a strong linear correlation was created between the water content and the soluble myofibrillar and sarcoplasmic proteins. The results suggested that these proteins may influence water loss. Other researchers have reported that the process of dehydration and chemical degradation of proteins and fats during the dry-aging process gives the dry-aged meat an intense aroma of walnut and beef, improving its taste and aroma [49], similar to the results observed in the given work.

Texture properties are a complex attribute, characterized by a large number of indicators, and which depend on various factors, such as chemical composition; structure; different muscle components, especially myofibrillar and connective tissues; physical properties; processing methods; form; and others [44,66,67]. In order to assess the specific characteristics of the texture, different measurements are performed on fresh or aged meat, especially to determine the tenderness, cohesiveness, hardness, and elasticity.

Changes in the meat tenderness of raw beef were reported in several studies [68,69], and after 24 h post-mortem, an increase in tenderness was observed as a result of the enzymatic degradation of muscle tissue. This degradation is caused by proteolytic enzymes and pH, the amount and degree of cross-linking of connective tissue, and animal species.

Table 3 shows the dynamics of the change in the texture profile analysis parameters (hardness, springiness, gumminess, and chewiness) of beef during dry-aging for 35 days.

The hardness of the meat is mainly influenced by the protein and water content. Collagen is identified as a determinant of textural differences in meat. Collagen molecules are linked together by intermolecular bonds that can provide structure and strength to the muscle [70]. During aging, the solubilization of collagen reduced the meat hardness.

Weston, et al. and Ripoll, et al. [70,71] suggested that raw meat is harder because of the viscous liquid that is filled between the fibers and bundles of fibers, and, in aged meat, the hardness is decreased due to changes in sarcoplasmic proteins. In the present study, hardness decreased in the first 14 aging days by 68.57%, 21 days—79.10%, 28 days—80.50%, and at the end of dry-aging period by 85.90%. These changes in the meat structure led to an increase in the springiness of the meat during aging [71], and a lower increase was observed in the first 21 days of aging of about 6.7%, and a double value was observed at the end of the dry-aging process—13.75%.

Chewiness is related to hardness, elasticity, and cohesiveness, and the values of chewiness decreased in meat subjected to the dry-aging process for 21 days by 82.78%, 28 days—83.7%, and 35 days—88.57%. Gumminess is related to hardness and cohesiveness, and the values of gumminess decreased in dry-aged beef by 83.86% in the first 21 days, about 85% in 28 days, and 89.95% in 35 days.

During aging, the cohesiveness of the meat decreased by 28.65% during the dry-aging period, and, therefore, the meat internal bond strength decreased due to the solubilization of the proteins, which could be the reason for its fragile structure. A similar pattern was observed by [72,73,74].

Similar results were obtained by Lee, et al. [55], where during the dry-aging for 63 days, hardness decreased from 19.4–34.70%, springiness increased non-essentially, gumminess decreased with a maximum of 49.5%, chewiness decreased by 80%, and cohesiveness decreased by 27%. Moreover, similar results were presented by Maqsood, et al. [63] in their paper.

The texture of the aged beef can be measured and expressed by Warner–Bratzler Shear Force (WBSF) parameters. Several authors showed that the WBSF of dry-aged beef decreased significantly with an increased dry-aging period, a process which is also due to the degradation/solubilization of meat proteins under the influence of various factors/action of the meat’s own enzymes [56,62]. Thus, using this method, Obuz, et al. [56] obtained a decrease of WBSF of 12–14%, and Kim, et al. [62] obtained a decrease of 23%.

**Figure 2:**
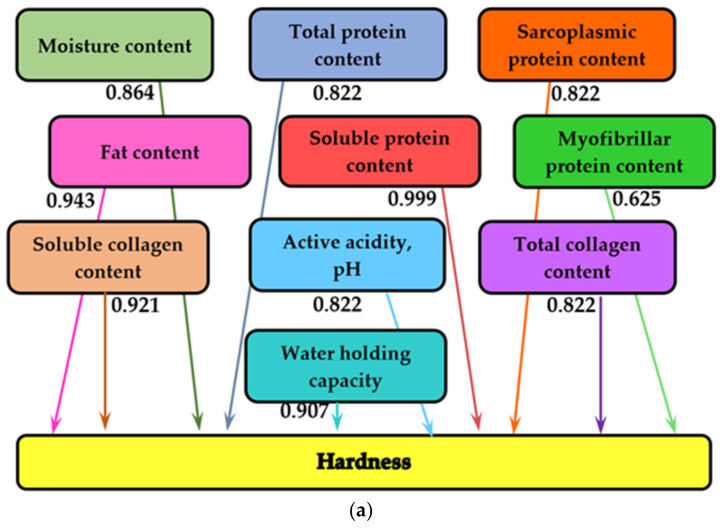

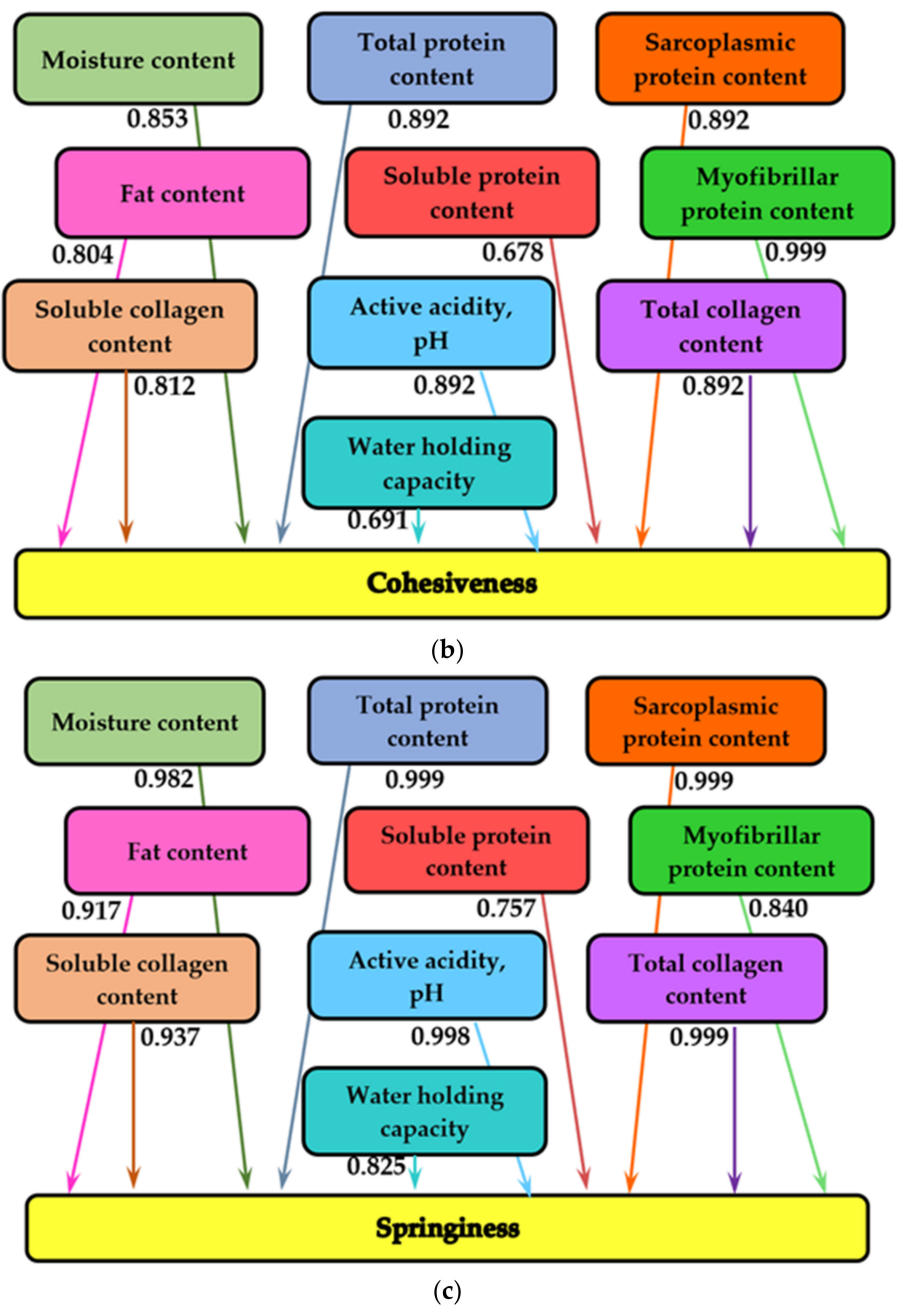

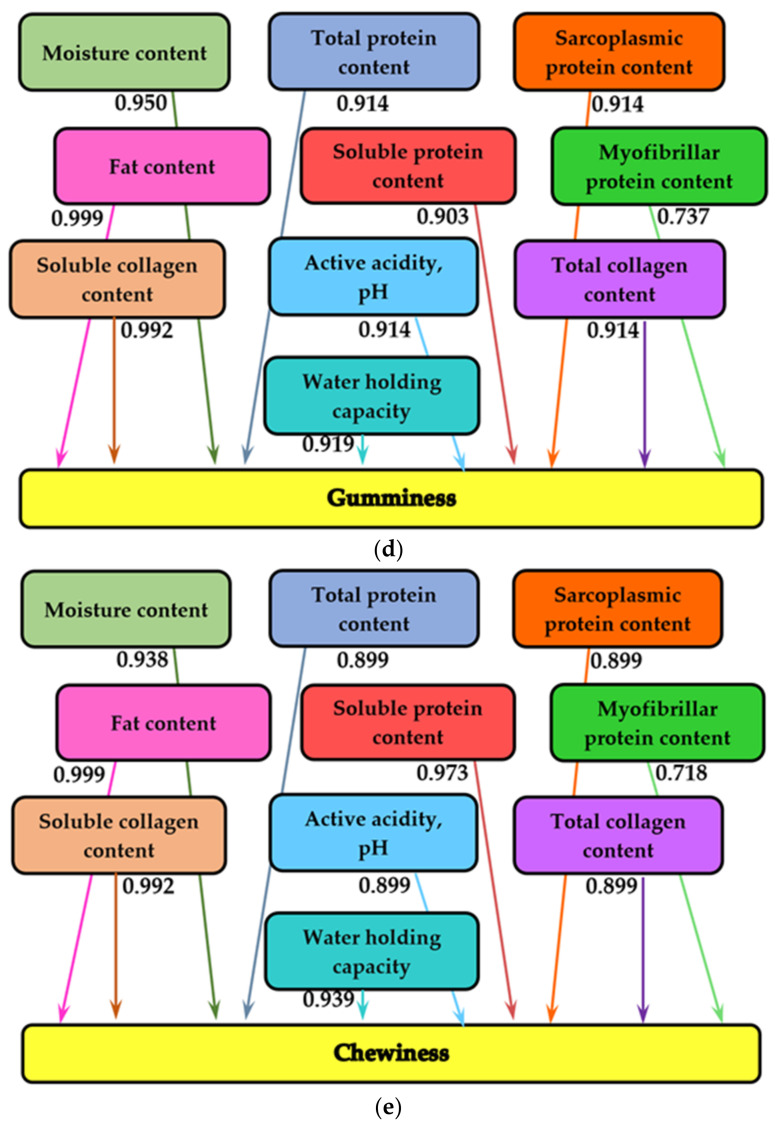
Analysis of mutual information on the influence of physicochemical indicators on texture profile parameters: (**a**) hardness; (**b**) cohesiveness; (**c**) springiness; (**d**) gumminess; (**e**) chewiness.

Variations in organoleptic and physicochemical parameters occur simultaneously during dry-aging and storage. In order to assess the measure of the influence of storage time on texture characteristics, with a significant impact on consumer perception, the method of information analysis was applied. The analysis of mutual information was applied in the study of the influence of different concentrations of rosehip powder and sea buckthorn flour on the physicochemical and organoleptic parameters of gingerbread and wheat bread [75,76]. Figure 2 shows the analysis of mutual information on the influence of physicochemical indicators on the texture profile parameters (hardness, cohesiveness, springiness, gumminess, and chewiness) in dry-aged beef for 35 days.

It is noted that hardness is largely influenced by soluble protein content (mutual information 0.999 bits), fat content (0.943 bits), soluble collagen content (0.921 bits), and *WHC* (0.907 bits). Total protein content and active acidity equally influence hardness in meat (0.822 bits). Hardness is also equally influenced by sarcoplasmic protein content and total collagen content (0.822 bits). Myofibrillar protein content has the least influence on the hardness of aged meat (0.625 bits) (Figure 2a).

In the case of cohesiveness, myofibrillar protein content influences this parameter to the greatest extent (0.999 bits). Total protein content, sarcoplasmic protein content, and total collagen content equally influence the cohesiveness of aged meat (0.892 bits). *WHC* influences cohesiveness to a lesser extent, with the value of the mutual analysis being (0.691 bits) (Figure 2b).

Similarly, Figure 2c shows a graph with the results of the information analysis for springiness, where the influencing factors are also the physicochemical indicators of meat quality that change over 35 days. Springiness is shown to be most strongly and equally influenced by total protein content, sarcoplasmic protein content, and myofibrillar protein content (0.999 bits). This texture parameter is also strongly influenced by active acidity (0.998 bits), moisture content (0.982 bits), soluble collagen content (0.937 bits), and fat content (0.917 bits). The soluble protein content has the least influence on the springiness of aged meat (0.757).

In the case of gumminess, fat content and soluble collagen content have the dominant influence (respectively, 0.999 bits and 0.992 bits), followed by moisture content (0.950 bits). Total protein content, sarcoplasmic protein content, active acidity, and total collagen content equally influence the gumminess of aged meat (0.914 bits). As with hardness, myofibrillar protein content has the least influence on meat gumminess (0.737 bits) (Figure 2d).

Chewiness is largely influenced by fat content (0.999 bits), soluble collagen content (0.992 bits), and soluble protein content (0.973 bits), followed by *WHC* (0.939 bits) and moisture content (0.938 bits). This parameter is equally influenced by total protein content, sarcoplasmic protein content, active acidity, and total collagen content (0.899 bits). As with hardness and gumminess, myofibrillar protein content has the least influence on the chewiness of aged meat (0.718 bits) (Figure 2e).

## 4. Conclusions

During the dry-aging of beef, a decrease in humidity was observed in the first 21 days, which allowed the concentration of fat, total protein, and total collagen. The dry-aging process influenced the meat pH value, which, in the second part of the dry-aging process (14–35 days), increased from 5.49 to 5.66. These values considerably favored the water-holding capacity (by 37.33%), and induced the activation of the meat’s own enzymes (calpain, cathepsin, collagenase), with a positive impact on the solubilization process of meat proteins and collagen during dry-aging. All of these factors influenced the solubilization process of proteins and collagen, contributing to the improvement of the beef texture profile parameters.

In order to highlight the influence of the physicochemical indicators on the texture profile parameters (hardness, cohesiveness, springiness, gumminess, and chewiness) on the dry-aged beef for 35 days, an analysis of the information was applied.

It was found that the hardness of the texture is largely influenced by the content of soluble proteins, fats, soluble collagen, and water-holding capacity. The total protein content, sarcoplasmic protein content, collagen, and active acidity have a moderate influence, whereas the myofibrillar protein content has a lesser influence on the hardness of the beef texture dry-aged for 35 days.

Myofibrillar protein content is the predominant factor in the cohesiveness of dry-aged beef, whereas the influence of other factors can be considered moderate. Springiness is strongly and equally influenced by the total protein content, the sarcoplasmic and myofibrillar protein content, as well as the active acidity, moisture, soluble collagen, and fat content. Chewiness and gumminess are mainly influenced by the content of fats, soluble proteins, and soluble collagen, followed by the content of water-holding capacity and moisture. Myofibrillar protein content has a minor influence.

The obtained results allow the optimization of the beef dry-aging technological process in order to obtain a product with a sensory profile preferred by the consumer.

## Figures and Tables

**Table 1:** Sensory characteristics for the evaluation of the finished product.

Sensory Characteristics	Product Description
Appearance	Wet surface, elastic muscle tissue, regular edges, no deep cuts.
Color	The color of the muscles is dark red, and the color of the adipose tissue is white without signs of rancidity, specific to beef.
Consistency	Elastic, firm, the dimple disappears faster.
Flavor	The characteristic flavor of beef is not perceived, a balanced aroma characteristic of matured meat, without a foreign smell.

**Table 2:** Sensory and physicochemical indicators of dry-aged beef (the results are presented as means ± standard deviation).

Indicators	Samples				
	Fresh Beef	M14	M21	M28	M35
Sensory indicators					
Average score of sensory profile	4.15 ± 0.05 ^a^	4.43 ± 0.05 ^b^	4.80 ± 0.04 ^c,d^	4.92 ± 0.05 ^d,e^	5.00 ± 0.00 ^e^
Appearance	4.26 ± 0.04 ^a^	4.66 ± 0.03 ^b^	4.92 ± 0.01 ^c^	4.95 ± 0.03 ^c^	5.00 ± 0.00 ^c^
Color	4.30 ± 0.04 ^a^	4.57 ± 0.04 ^b^	4.88 ± 0.03 ^c^	4.92 ± 0.03 ^c,d^	5.00 ± 0.00 ^d^
Consistency	4.05 ± 0.03 ^a^	4.36 ± 0.02 ^b^	4.82 ± 0.05 ^c,d^	4.95 ± 0.03 ^d^	5.00 ± 0.00 ^d^
Flavor	4.00 ± 0.01 ^a^	4.13 ± 0.03 ^b^	4.57 ± 0.01 ^c^	4.84 ± 0.02 ^d^	5.00 ± 0.00 ^e^
Physicochemical indicators					
Moisture content, %	59.55 ± 0.45 ^d^	55.98 ± 0.55 ^c^	55.80 ± 0.37 ^c^	54.67 ± 0.38 ^b^	53.31 ± 0.16 ^a^
Activ acidity pH, c.u.	5.50 ± 0.01 ^a^	5.54 ± 0.01 ^b^	5.56 ± 0.01 ^b^	5.65 ± 0.01 ^c,d^	5.66 ± 0.01 ^c,d^
Water holding capacity, %	66.41 ± 0.41 ^a^	84.42 ± 0.98 ^b^	91.11 ± 0.40 ^c^	91.17 ± 0.46 ^c^	91.20 ± 0.49 ^c^
Fat content, %	6.40 ± 0.20 ^a^	8.56 ± 0.07 ^b^	9.02 ± 0.18 ^b,c^	9.34 ± 0.21 ^c^	9.50 ± 0.12 ^c^
Total collagen content, %	0.41 ± 0.010 ^a^	0.47 ± 0.006 ^b^	0.48 ± 0.005 ^b^	0.52 ± 0.004 ^c^	0.52 ± 0.006 ^c^
Soluble colagen content, %	0.05 ± 0.004 ^a^	0.11 ± 0.006 ^b^	0.12 ± 0.005 ^b,c^	0.12 ± 0.003 ^b,c^	0.13 ± 0.002 ^c^
Protein content, %	24.64 ± 0.07 ^d^	23.78 ± 0.36 ^c,d^	23.59 ± 0.45 ^b,c,d^	22.99 ± 0.34 ^a,b,c^	22.79 ± 0.36 ^a^
Soluble protein content, %	2.61 ± 0.02 ^a^	2.98 ± 0.05 ^b^	2.99 ± 0.01 ^b^	3.00 ± 0.01 ^b^	3.01 ± 0.004 ^b^
Sarcoplasmatic protein content, %	1.22 ± 0.02 ^a^	1.59 ± 0.06 ^b^	1.68 ± 0.05 ^b,c^	1.79 ± 0.03 ^c^	1.99 ± 0.02 ^d^
Miofibrilar protein content, %	1.39 ± 0.03 ^d^	1.38 ± 0.04 ^c,d^	1.32 ± 0.03 ^c^	1.10 ± 0.03 ^a,b^	1.02 ± 0.02 ^a^

Different letters (^a–d^) designate statistically different results (*p* ≤ 0.05). M14—dried beef for 14 days; M21—dried beef for 21 days; M28—dried beef for 28 days; M35—dried beef for 35 days.

**Table 3:** Dynamics of change in texture parameters of beef during dry-aging process (results are presented as averages ± standard deviation).

Texture Parameters	Samples				
	Fresh Beef	M14	M21	M28	M35
**Hardness, g**	1592.55 ± 48.11 ^c^	500.53 ± 13.75 ^b^	331.58 ± 34.87 ^b^	310.77 ± 40.12 ^b^	224.12 ± 32.98 ^a^
**Cohesiveness, %**	0.520 ± 0.006 ^d^	0.426 ± 0.008 ^c^	0.403 ± 0.008 ^b^	0.400 ± 0.007 ^b^	0.371 ± 0.009 ^a^
**Springiness, %**	0.705 ± 0.004 ^a^	0.745 ± 0.007 ^b^	0.752 ± 0.006 ^b^	0.766 ± 0.002 ^c^	0.802 ± 0.001 ^d^
**Gumminess, g**	828.13 ± 28.56 ^c^	231.22 ± 21.98 ^b^	133.63 ± 10.24 ^a^	124.31 ± 4.12 ^a^	83.15 ± 1.89 ^a^
**Chewiness, g**	583.83 ± 12.04 ^c^	158.85 ± 10.47 ^b^	100.48 ± 13.54 ^a^	95.21 ± 11.57 ^a^	66.68 ± 4.23 ^a^

Different letters (^a–d^) designate statistically different results (*p* ≤ 0.05). M14—dried beef for 14 days; M21—dried beef for 21 days; M28—dried beef for 28 days; M35—dried beef for 35 days.

## Data Availability

No new data were created or analyzed in this study. Data sharing is not applicable to this article.
